# Demographics, treatment trends, and survival rate in incident pulmonary artery hypertension in Korea: A nationwide study based on the health insurance review and assessment service database

**DOI:** 10.1371/journal.pone.0209148

**Published:** 2018-12-19

**Authors:** Shinjeong Song, Sang-Eun Lee, Sang Kwon Oh, Seong A. Jeon, Ji Min Sung, Jae-Hyeong Park, Hyuk-Jae Chang

**Affiliations:** 1 Division of Cardiology, Severance Cardiovascular Hospital, Yonsei University College of Medicine, Yonsei University Health System, Seoul, South Korea; 2 Yonsei-Cedars-Sinai Integrative Cardiovascular Imaging Research Centre, Yonsei University College of Medicine, Yonsei University Health System, Seoul, South Korea; 3 Member of Healthcare Review and Assessment Committee, Health Insurance Review Assessment Service, Seoul, South Korea; 4 Department of Cardiology in Internal Medicine, Chungnam National University Hospital, Chungnam National University College of Medicine, Daejeon, Korea; 5 Yonsei Cardiovascular Research Institute Yonsei University College of Medicine, Yonsei University Health System, Seoul, South Korea; Kurume University School of Medicine, JAPAN

## Abstract

Epidemiologic data regarding pulmonary arterial hypertension (PAH) have relied on registries from Western countries. We assessed the current status of PAH in the Korean population. The Health Insurance Review and Assessment Service (HIRA) claim database, which comprises nationwide medical insurance data of Koreans from 2008–2016, was assessed to determine the current status of PAH. Overall, 1,307 patients were newly diagnosed with PAH from 2008–2016 (0.0005%, annual incidence: 4.84 patients/1 million people/year). The mean age at diagnosis was 44±13 years (range 18–65) and patients were mostly women (n = 906, 69.3%). Cases of idiopathic PAH (51.6%) accounted for the largest proportion, followed by acquired PAH (APAH) associated with congenital heart disease (25.8%) and APAH with connective tissue disease (17.2%). Overall, 807 (61.7%) patients received a single PAH-specific treatment based on their last prescription, of which bosentan (50.6%) was the most frequently used. Only 240 (18.4%) patients received combination therapy, with the bosentan-beraprost combination (32.9%) being the most common. During the mean follow-up of 1.9 years, the 1-, 2-, 3-, and 5-year estimated survival rates were 85%, 62%, 54%, and 46%, respectively. The prevalence and incidence of PAH in the Korean population is currently comparable with that in previous registries. The 5-year survival rate was slightly higher in the Korean population than previously reported.

## Introduction

Pulmonary arterial hypertension (PAH) is a disease characterized by remodeling of the small pulmonary arteries, which leads to a progressive increase in pulmonary vascular resistance and right-sided heart failure and death.[1–6] Over the past decade, PAH-specific drugs such as prostanoids, endothelin receptor antagonists (ERA), and phosphodiesterase-5 inhibitors (PDE5-i) that can alter the natural history of PAH, have been developed [7–11]; consequently, albeit not satisfactorily, clinical outcome of PAH has also improved.[12–14]

Several PAH registries including the Korean Registry of Pulmonary Arterial Hypertension (KORPAH) have provided basic information on PAH in terms of its epidemiology, clinical characteristics, and prognosis.[15–18] However, most data have derived from registries of Western populations, thus leaving the Asian population incompletely evaluated. Further, the natural history of PAH in the era of PAH-specific drugs and a comprehensive investigation of the use of PAH-specific medications have not been adequately investigated.

Therefore, we assessed the prognosis and the pattern of using PAH-specific drugs in incident PAH patients, using the Korean Health Insurance Review and Assessment Service (HIRA) database of medical claims.

## Methods

### National Health Insurance data of South Korea

We used data derived from the HIRA database, which consists of all medical expenses claim data of South Koreans between 2007 and 2016 with a mean follow-up of 1.9 years.

The HIRA contains the medical billing data of the entire South Korean population (97% health insurance, 3% medical care). In the billing statement, identifiers for the prescriber, and the number of visits, number of days spent in outpatient visits, number of prescriptions (total medical care costs, the partial deductible for an insured person, insurer’s contribution), details of medical treatment items (classified by mechanism of action, drug, and treatment material), and details of prescribed prescriptions (prescription and preparation details for each drug) are all included.

The HIRA also contains information regarding medical history (treatments, procedures, examinations), diagnoses, and costs of procedures or drugs. Also, all HIRA data were fully anonymized before we accessed them. The institutional review board of the Severance Hospital at the Yonsei University College of Medicine in Seoul, Republic of Korea approved this study (No. 4-2017-0073). The institutional review board waived the requirement to obtain informed consent.

### Selection of incident pulmonary arterial hypertension patients

To estimate the incidence of PAH in the Korean population, Korean adults diagnosed with PAH, aged 18–65, between 2007 and 2016 were first identified within the HIRA database. Over this period, total Korean population aged 18–65 years was 35,927,664.

PAH was defined using the International Classification of Diseases-Tenth Revision (ICD-10) codes and prescription of PAH-related medications, as echocardiography or right-side catheterization results were not included in the HIRA database. Patients with PAH were defined as subjects meeting the following two criteria: 1) subjects with a diagnosis defined by ICD-10 codes for PAH (I27.0 and I27.2) and 2) subjects with at least one prescription of one or more of the following drugs: a calcium channel blocker (CCB) (nifedipine, amlodipine, and diltiazem) and a PAH-specific drug (prostanoids, ERA, and PDE5-i). Among those with a record in the HIRA database, 14,255 patients had ICD I27.0 and I27.2 codes, of which 5,034 had a PAH-related prescription. To determine the incidence of PAH and identify newly diagnosed patients, we excluded all patients with a record of a hospital visit and prescriptions between January 1, 2007 and December 31, 2007.

As the ICD I27.0 and I27.2 codes indicate a diagnosis of pulmonary hypertension as well as PAH. To extract only information on PAH, idiopathic PAH (IPAH) and acquired PAH (APAH) were defined and extracted separately. We defined IPAH as having a code of I27.0 with no codes related to connective tissue disease (CTD), congenital heart disease (CHD), portal hypertension, human immunodeficiency virus (HIV), schistosomiasis, or chromic hemolytic anemia. Several IPAH diagnostics were included in the ICD I27.2, particularly in the subgroup I27.2 diagnosis, where pulmonary hypertension (PH) Group 2, 3, 4, and 5 diagnoses are included in addition to PAH (Group 1). Therefore, in order to screen only patients with pure IPAH, patients with a left-side heart disease related diagnosis, lung disease, and/or hypoxia (chronic respiratory disease, chronic thromboembolic PH, and other pulmonary artery obstruction related ICD-10 codes are recorded in the Supplementary) were excluded. Patients with a diagnosis of PH group 5 were also excluded.

As for APAH, we attempted to classify APAH as CTD, CHD, by ICD-10 codes related to the disease (the ICD-10 codes used are described in S1 Table).

Other comorbidities (definitions are indicated in S2 Table), such as hypertension and diabetes, were also identified from the medical claims records according to the ICD-10 codes. The following baseline demographics were collected: age at diagnosis, sex, specific etiology of PAH, prescription medication(s), comorbid condition(s), and examination modalities (transthoracic echocardiography, right heart catheterization [RHC], and cardiac magnetic resonance imaging).

Drug codes were identified through drug nomenclature of prostanoids, ERAs, PDE5-i, and CCB registered in the HIRA data. PAH-specific monotherapy or combination therapy was analyzed based on the last prescribed drug for each patient. We also investigated changes in the use of medications, as it was assumed that for some patients medications changed different reasons (worsening symptoms, side effects, etc.).

### Primary endpoint

The primary outcome was all-cause death. As the claims database contains clinical results and classification codes, we identified deaths in the hospital using this code. Additionally, considering that the code for hospital death could be underestimated, people with no claims data for over one year were regarded as dead. The types of PAH-specific drugs, types and frequency of examinations related to PAH, number of hospitalizations, and length of hospital stay were analyzed. Events of all cause in-hospital mortality according to the etiology, age, and prescription medication were surveyed.

### Statistical analysis

To describe the baseline characteristics and co-morbid conditions of all PAH patients and incident PAH cases, we used frequencies (proportions) for categorical variables and means±standard deviations (SDs) for continuous variables. To compare patients’ baseline characteristics according to PAH subgroups (IPAH or APAH), we used the independent *t*-test or the Wilcoxon rank-sum test for continuous variables and the chi-square test for categorical variables. Survival rates from enrolment of newly diagnosed patients with PAH were calculated using the life-table method. Log-rank tests were used to compare survival rates according to PAH subgroups (IPAH, CHD, and CTD), age, and types of medication. All-cause mortality according to the etiology, age, and prescription medication were analyzed using Kaplan-Meier survival analysis. P value of <0.05 was considered statistically significant.

## Results and discussion

### Demographic and baseline characteristics of all patients

**Fig 1:**
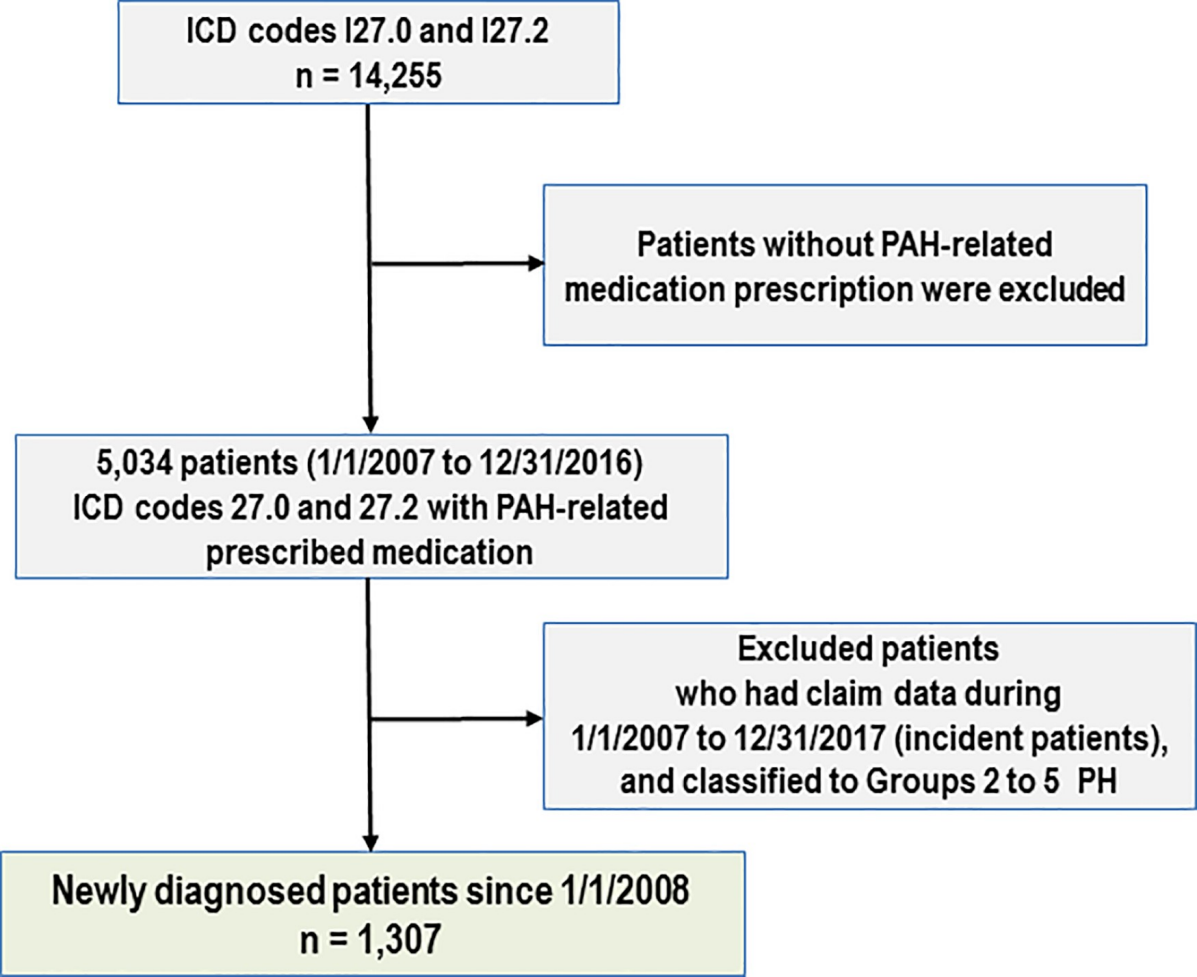
Inclusion flow diagram of newly diagnosed pulmonary arterial hypertension patients enrolled in the study. PAH, pulmonary arterial hypertension; ICD-10, International Classification of Diseases-Tenth Revision.

**Fig 2:**
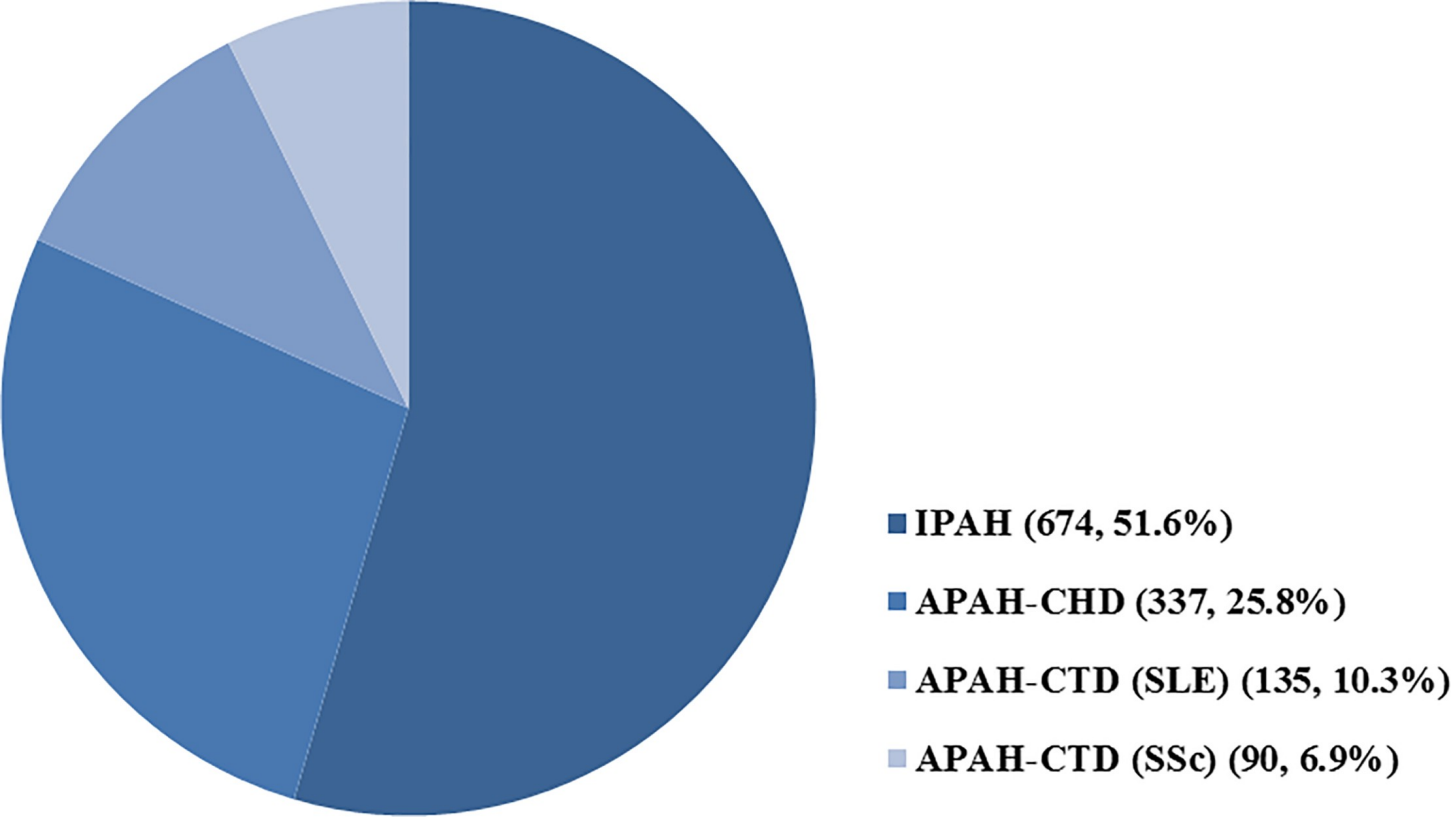
Pulmonary arterial hypertension etiological breakdown of patients at enrolment.

From January 2008 to December 2016, we identified 1,307 patients diagnosed with PAH (ICD codes I27.0x and I27.2x) for the first time with at least one drug prescription (CCB, prostacyclin analogues, ERA, or PDE5-i) (Fig 1). The baseline characteristics and comorbid conditions of all patients and their respective percentages according to the specific etiologies are summarized in Table 1. The mean age at enrolment was 44±13 years for all 1,307 patients. There were 906 female patients (69.3%). The most common etiology of PAH was idiopathic (n = 674, 51.6%), followed by CHD (n = 337, 25.7%) and CTD (n = 296, 22.6%) (Fig 2). Approximately 30.0% of patients had essential hypertension and 5.8% had diabetes.

**Table 1:** The baseline characteristics and comorbid conditions of all patients and their respective percentages according to the specific etiologies.

	All patients	IPAH group	APAH group	P-value	APAH-CHD	APAH-CTD-SSc	APAH-CTD-SLE	APAH-CTD-other
Patients, n (%)	1307 (100.0)	674 (51.6)	633 (48.4)		337 (53.2)	90 (14.2)	135 (21.3)	71 (11.2)
Age	44±13	48±12	41±12	<0.001	40±12	50±10	38±11	43±12
Female sex, n (%)	906 (69.3)	409 (60.7)	497 (78.5)	<0.0001	218 (64.7)	86 (95.6)	133 (98.5)	60 (84.5)
Comorbidity								
HTN, n (%)	392 (30.0)	285 (42.3)	107 (16.9)	<0.0001	44 (13.1)	18 (20.0)	34 (25.2)	11 (15.5)
DM, n (%)	76 (5.8)	55(8.2)	21 (3.3)	0.0002	10 (3.0)	5 (5.6)	3 (2.2)	3 (4.2)
Renal failure (CKD), n (%)	32 (2.5)	22 (3.3)	10 (1.6)	0.0489	1 (0.3)	3 (3.3)	9 6.7)	0 (0.0)
Arrhythmia, n (%)	81 (6.2)	46 (6.8)	35 (5.5)	0.3316	1 (0.3)	0 (0.0)	1 (0.7)	2 (2.8)
TIA or stroke, n (%)	29 (2.2)	19 (2.8)	10 (1.6)	0.1285	7 (2.1)	0 (0.0)	2 (1.58)	1 (1.4)
Follow-up duration (year)	2.7±2.2	2.3±2.0	3.1±2.4	<0.0001	3.1±2.4	3.0±2.3	3.4±2.5	3.2±2.4

IPAH, idiopathic pulmonary arterial hypertension; APAH, acquired pulmonary arterial hypertension; HTN, hypertension; DM, diabetes mellitus; IHD, ischemic heart disease; CKD, chronic kidney disease; TIA, transient ischemic attack

The incidence trend of PAH is shown in S1 Fig. Approximately 4 people per million people were diagnosed as having PAH annually. In the incident cases, the distribution of patients by age and the average follow-up duration are as follows. Based on the 2016 population of 18 to 65 years old, the incidence of PH was 4.8 per 1 million and the prevalence was 20.2 per 1 million.

### Primary endpoint: All-cause death

**Fig 3:**
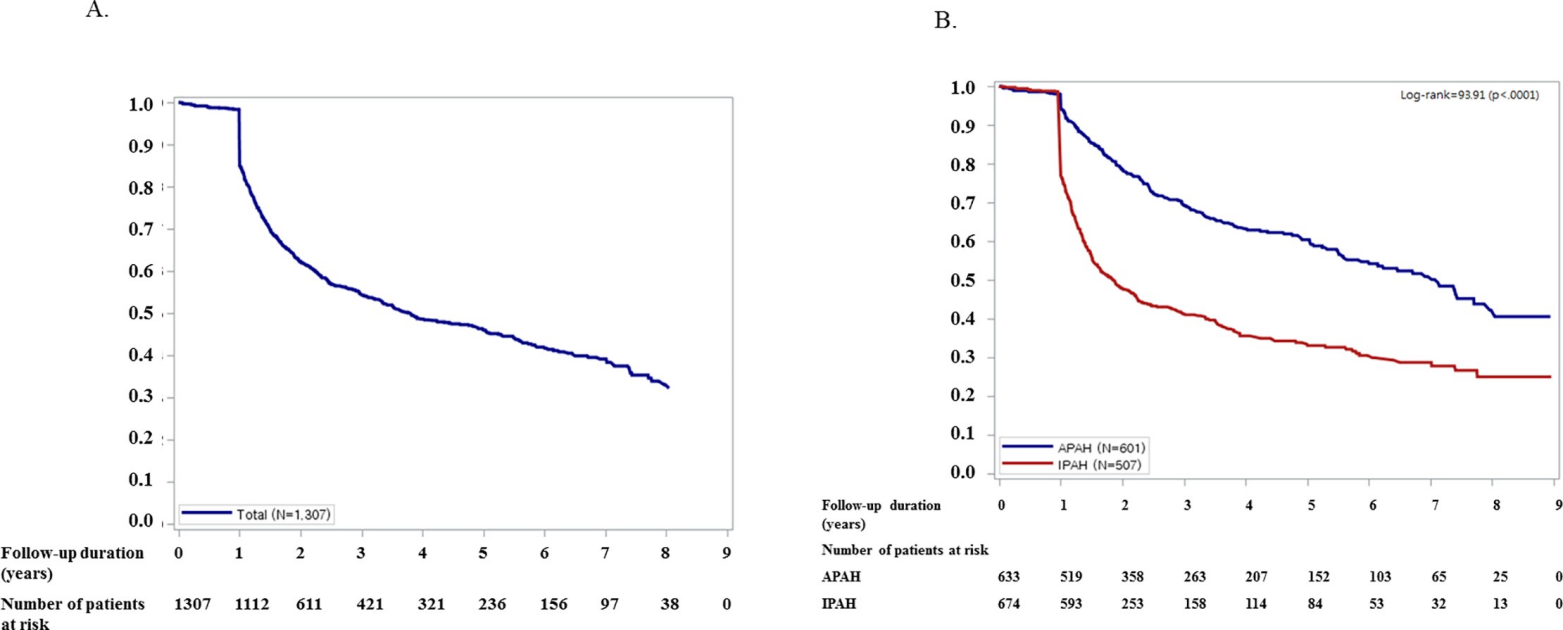
Cumulative survival curve of patients with pulmonary arterial hypertension. (A) The 1-year, 2-year, and 3-year estimated survival rates shown are 84.9%, 62.2%, and 54.3%, respectively. (B). Primary outcome: cumulative survival curve of patients with pulmonary arterial hypertension according to etiology. IPAH, idiopathic pulmonary arterial hypertension; APAH, acquired pulmonary arterial hypertension.

From January 1, 2008 to December 31, 2016, of the 1,307 incident cases, 581 cases (44.5%) of mortality occurred during the mean 1.9-year follow-up. The cumulative survival curve of incident cases is presented in Fig 2. After a mean (±SD) follow-up of 1.9±1.5 years, the estimated survival rates at 1 year, 2 years, 3 years, and 5 years were 84.9%, 62.2%, 54.3%, and 46.0%, respectively (Fig 3A). Fig 3B presents the comparison of prognosis according to the etiologies of PAH. IPAH patients had the highest mortality, followed by patients with CTD and CHD (P = 0.001).

### Distribution and trend of prescribed medications

Based on the last medication prescribed to each patient, 80.1% received specific treatments, of which only 22.9% received PAH-specific monotherapy and 76.8% received PAH-specific combination therapy (Table 2). In patients with APAH, bosentan was the most frequently used drug. There was a significant difference between monotherapy and combination therapy in patients with IPAH and those with APAH. The use of PAH target drugs was more frequent in patients with APAH than in those with IPAH.

**Table 2:** The distribution of drug monotherapy and combination therapy in patients with pulmonary arterial hypertension.

Specific drug therapy	All patients (n = 1307)	IPAH group (n = 674)	APAH group (n = 633)	P-value	APAH-CHD (n = 337)	APAH-CTD (n = 296)	P-value
Monotherapy n, (%)	807 (61.7)	397 (58.9)	410 (64.8)	<0.0001	225 (66.8)	185 (62.5)	0.0003
Bosentan, n, (%)	408 (50.6)	121(30.5)	287 (70.0)	<0.0001	174 (77.3)	113 (61.1)	<0.0001
Beraprost, n, (%)	284 (35.2)	219 (55.2)	65 (15.9)		22 (9.8)	43 (23.2)	
Sildenafil, n, (%)	58 (7.2)	25 (6.3)	33 (8.0)		23 (10.2)	10 (5.4)	
Ambrisentan, n, (%)	36 (4.5)	20 (5.0)	16 (3.9)		2 (0.9)	14 (7.6)	
Macitentan, n, (%)	15 (1.9)	8 (2.0)	7 (1.7)		3 (1.3)	4 (2.2)	
Iloprost, n, (%)	6 (0.7)	4 (1.0)	2 (0.5)		1 (0.4)	1 (0.5)	
Combination therapy n, (%)	240 (18.4)	74 (11.0)	57 (9.0)	<0.0001	96 (28.5)	70 (23.7)	0.0003
Beraprost-Bosentan, n, (%)	79 (32.9)	13 (17.6)	66 (39.8)	<0.0001	30 (31.3)	36 (51.4)	0.0035
Bosentan-Sildenafil, n, (%)	43 (17.9)	8 (10.8)	35 (21.1)		27 (28.1)	8 (11.4)	
Macitentan-Sildenafil, n (%)	43 (17.9)	19 (25.7)	24 (14.5)		17 (17.7)	7 (10.0)	
Beraprost-Bosentan-Sildenafil, n (%)	17 (7.1)	3 (4.1)	14 (8.4)		10 (10.4)	4 (5.7)	

IPAH, idiopathic pulmonary arterial hypertension; APAH, acquired pulmonary arterial hypertension; PAH, pulmonary arterial hypertension

**Fig 4:**
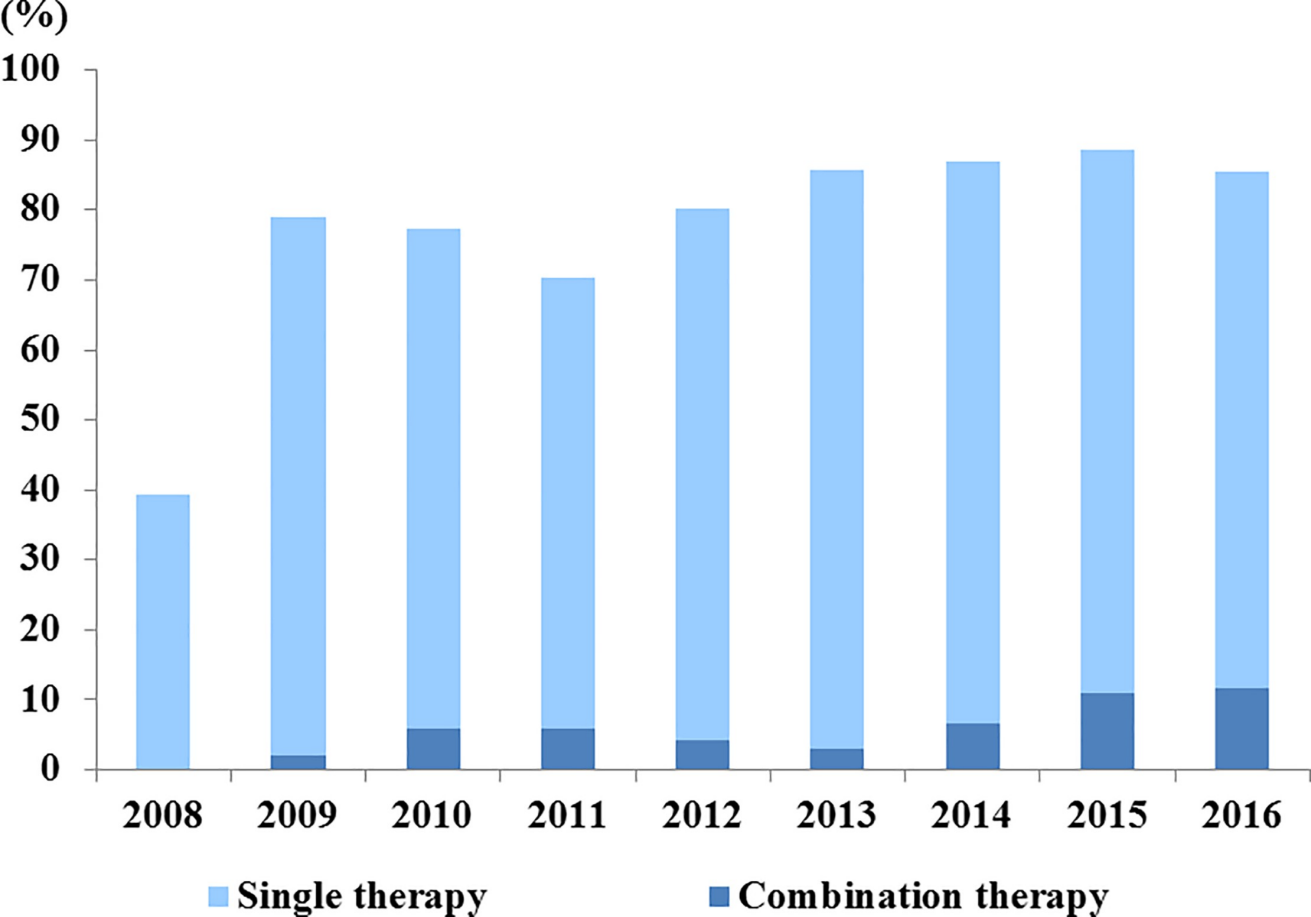
Distribution and trend of prescribed pulmonary arterial hypertension-specific drugs.

The rate of use of PAH-specific drugs has steadily increased every year in PAH patients. In 2009, the rate of subscribing PAH-specific drugs was 79%, compared with 40% in 2008. In addition, the rate of prescribing PAH-specific drug combinations increased to 11% (Fig 4).

### Right-sided catheterization

**Fig 5:**
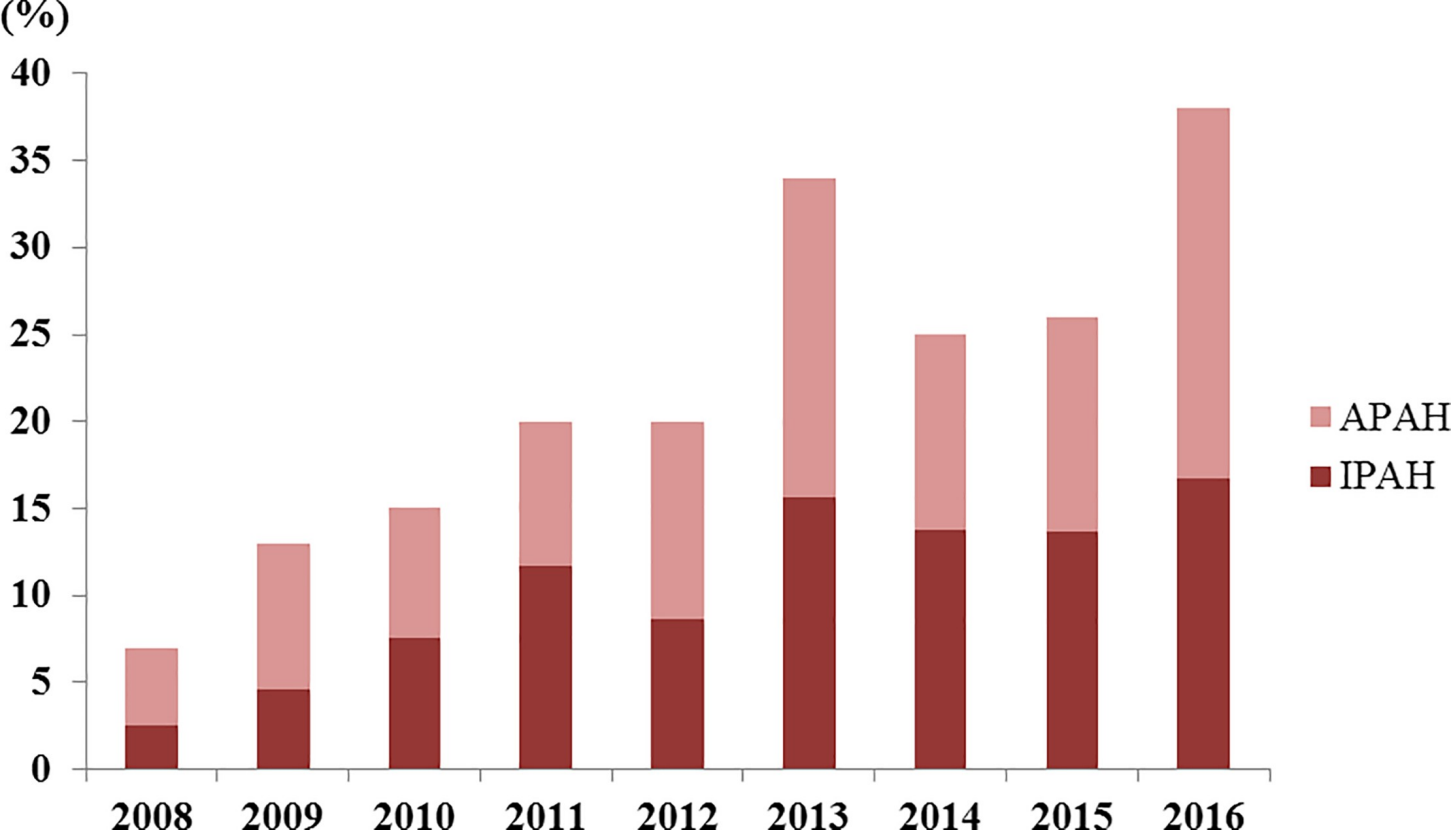
The trend of right catheter catheterization according to etiology in newly diagnosed patients per year. IPAH, idiopathic pulmonary arterial hypertension; APAH, acquired pulmonary arterial hypertension.

Right-sided catheterization (RHC) was performed in 328 patients (25.1%), which comprised 22.4% (151 patients) with IPAH and 28.0% (177 patients) with APAH. The proportion of newly diagnosed patients who underwent RHC was 7% in 2008, but it has increased continuously since then; in 2016, RHC was performed in 38% cases. (Fig 5) There were no significant differences in the rate of RHC tests based on etiology; for both IPAH and APAH, the rates of RHC examination increased.

### Hospitalization and costs

The percentage of patients who were admitted more than once was 55.1% (n = 720). The average duration of admission was 9±7 days. The average annual total amount of medical care benefits per patient was 1,782,000 won ($16,608), and average annual deductible medical cost per patient was 1,770,000 won ($1,649).

## Discussion

The present study is the first report of clinical characteristics and epidemiology of all PAH patients in Korea. The important findings were as follows: 1) the most common etiology of PAH was IPAH, followed by CTD and CHD in incident cases; 2) female sex was predominant (69.3%); 3) patients concurrently had hypertension (30.0%) and diabetes (5.8%); 4) the incidence rate of PAH was 4.84 patients/1 million people; 5) the estimated mean follow-up duration was 1.9 years; and 6) 1-year, 2-year, and 3-year estimated survival rates were 85%, 63%, and 56% respectively.

Previous large national observational registries, including the KORPAH of Korea, have provided information on current PAH epidemiology, increasing awareness about the disease.[15–20] In the present study, it is significant that the total number of patients with PAH was investigated by using unique data on the medical expenses taking into consideration of the possibility missing unregistered patients in a previous national registry.

The demographic characteristics of the patients with PAH in the HIRA database were similar to those of the previous KORPAH registry participants. The observed mean age (44 years) in the HIRA database was similar to that of the KORPAH registry (47.6 years), and the proportion of female patients (69.3%) was less in the HIRA database than in the KORPAH registry (80.5%). In addition, the ratio of IPAH to APAH was similar to that in other registries, including the KORPAH. In the French and REVEAL registries, approximately half of the patients exhibited IPAH, whereas the other half exhibited APAH.[15–17] Systemic hypertension was the most commonly reported comorbid condition (30.0% of all patients, especially 42.3% in IPAH patients). Similarly, in the REVEAL registry, the average diagnostic age of patients with IPAH was 49.9 years and 80.3% of women, but the proportion of hypertension was 41.8%. In this study, authors describe the cause of systemic hypertension in PAH patients as either a generalized vasculopathy or common substrate affecting both the pulmonary and systemic circulations, such as sleep apnea or diastolic dysfunction. In other words, it is explained by other causes besides gender and aging which are considered to be common causes of hypertension.[15] In addition, several studies have described the cause of systemic hypertension in pulmonary hypertension as left ventricular diastolic dysfunction and heart failure of preserved ejection fraction (HFPEF).[21–23] Compared with previous registries, the most notable difference in the KORPAH registry and a limitation in the PAH patients of the HIRA database was the performance rate of RHC. The rate of RHC increased yearly, reaching about 40% in the last year, but overall among the 1307 patients, only 328 patients (25.1%) underwent RHC. RHC was performed slightly more frequently in APAH than in IPAH patients (28.0% vs. 22.4%, p = 0.02). PAH is diagnosed with RHC (mean pulmonary artery pressure (mPAP) ≥25 mmHg, pulmonary artery wedge pressure (PAWP) ≤15 mmHg, and pulmonary vascular resistance (PVR) >3 Wood units). Therefore, the unknown occurrence of RHC in the patients of this cohort and RHC performed in approximately 25% of patients may be a limitation to PAH validation. For example, there may be doubt as to whether post-capillary PH was adequately excluded, and right ventricular systolic pressure (RVSP) may have been overestimated on echocardiography performed for RHC. In the KOPARH registry as well as in this cohort, the underuse of RHC suggests the attitude of physicians towards the Korean PAH diagnosis. In fact, PAH diagnoses by cardiologists, who have easy to access to RHC, were approximately 50% half[18], while the remainder were diagnosed in rheumatology or pulmonology units. In Korea, the cost burden on medical expenses is relatively limited. Therefore, the proportion of physicians or patients who is reluctant to order further testing is low. In addition, due to disease severity at the time of diagnosis (in KORPAH, NYHA Class III, IV patients accounted for about 43%), the patient's condition may not have been suitable for performing RHC testing and an alternative test may have been performed. However, in order to exclude a PH of groups 2–5 including post-capillary PH, we excluded the corresponding diagnosis for each group. Therefore, the merits of this study were that pure, incident PAH patients were identified in nation-wide based database.

Another difference from other studies was the slightly higher proportion of IPAH (51.6%) patients than in other registries. This was likely due to the impossibility of distinguishing the exact diagnosis from the ICD-10 codes. As shown in S3 Table, IPAH and APAH diagnoses are combined in code I27.0. Also, I27.2 codes contain sub-diagnostic codes for both PH and PAH. In addition, it is believed that differential diagnostic tests (such as the pulmonary function test [PFT], RHC, chest high resolution computed tomography [HRCT], HIV testing, or autoimmune testing) were infrequent in patients diagnosed with IPAH.

Estimating mortality using ICD codes for hospital mortality is likely to be underestimated. The maximum number of prescription days available at medical institutions in Korea is 180 days. Because we determined that the number of days between hospital visits could be longer than the number of days prescribed depending on the patient's compliance, we defined death as having no medical claim for over one year. Therefore, compared to the real world, the one-year mortality rate may be relatively low and other mortality rates may be judged to be higher. Nonetheless, the survival rates at 1, 2, and 3 years were lower than those of other registries. In this regard, more specialized medical therapies are needed to improve the survival of patients with PAH in Korea. In addition, a follow-up study will need to investigate the outcome of pulmonary arterial hypertension-specific therapy. Recently, Tamura *et al*.[24] reported on the effectiveness and outcome of pulmonary arterial hypertension-specific therapy in Japanese patients with pulmonary arterial hypertension. This study will be helpful for follow-up studies.

The strength of this study was that we were able to identify the medications used to treat patients. Guidelines recommend the use of monotherapy of CCB, all licensed ERA drugs (ambrisentan, bosentan, and macitentan), and PDE5-i drugs (sildenafil and tadalafil), iloprost, and treprostinil as class I agents according to the World Health Organization functional class (WHO-FC).[25] Approximately 20% of patients received non-target therapy (CCB), 18% received PAH-specific monotherapy, and 62% received PAH-specific combination therapy. However, the proportion of PAH-specific therapies in patients who were followed up annually increased, and in particular, the proportion of patients given combination therapy gradually increased. We defined PAH as ICD codes I27.0 or I27.2 and use of medication for PAH. There may be questions about whether CCB is used in patients with hypertension (33.0% of the total patients) or arrhythmia (6.2% of the total patients), but not for PAH. Therefore, it may be a limitation of this study that there may have been an overestimation of patients who are prescribed CCB as a hypertension treatment for PH. However, in contrast, it means that the PH is not treated, and the inclusion of CCB drugs does not lead to overestimation, but rather it indicates the realities of patient treatment for hypertension. Considering that the proportion of patients who underwent conventional treatment in the KORPAH registry was about 27%, this cannot be judged as overestimated. As this cohort contains data regarding all drug prescriptions, further investigation in relation to the distribution of drug use, the trends of change, and the outcome of patients is expected.

Another strength of this study is that we enrolled patients with pure incident cases without referral bias (which reflects characteristics of patients referred to a specific hospital) and bias according to the practice pattern (subspecialty). This study overcomes referral bias with the use of nationwide registry covering all Korean patients. Therefore, patient characteristics (mortality rate, etc.) and comorbidity prevalence (HTN, etc.) of this study can be more accurately reflected in the real world. About 60% over of PAH patients received PAH-specific treatment in the KOPARH registry, meanwhile in this study, 80% patients received PAH-specific treatment. This difference can be explained by the practice pattern bias. In KORPAH, a large number of patients were enrolled based on echocardiography in a specific department (rheumatology department), and PAH-specific treatments were often not received in the department despite the diagnosis of PAH.

### Limitations

This study has some limitations. First, it was subject to all the limitations inherent to a retrospective cohort analysis. Second, certain baseline parameters were not recorded. Thus, we could not obtain information regarding clinical parameters (echocardiographic parameters, 6-minute walking distance, pulmonary vascular resistance, and WHO-FC) of patients with PAH. Therefore, prognosis according to the functional class could not be analyzed. Third, we validated PAH based on disease codes and medication prescribed. Prescriptions of CCB and specific agents (ERA, PDE5-i, and PG analogues) were used to validate patient diagnosis of PAH. However, CCB (diltiazem, amlodipine, and nifedipine) are also usually prescribed for blood pressure control in patients with hypertension and for heart rate control in patients with atrial fibrillation. The dosage of CCB for treating PAH is high; however, if we identified the prescribed dose for the patient, it was possible to accurately validate PAH cases. Because we could not confirm the prescribed dose, it is possible that PAH incidence is overestimated. Fourth, in case wherein the physician does not enter all the diagnoses in the database, classification of the etiology by the ICD code alone is not accurate. Particularly ICD codes I27.0 and I27.2 include mixed IPAH and APAH (diagnostic ICD codes I27.0 and I27.2 are described in S3 Table). Fifth, in-hospital death was confirmed by a specific code in the medical claim, but it was not known whether the death was related to PAH. If there was no claim for more than one year, it was considered death even if the cause of death was not confirmed. Although there was an advantage of being able to investigate the use of drugs in detail, the HIRA database does not include the WHO-FC, and thus, we could not assess the appropriateness of PAH-specific drug use according to the functional classification recommended by the guidelines. It cannot be determined whether appropriate medication was prescribed to suitable patients.

## Conclusions

We report the clinical data of 1,307 patients with PAH in Korea in this article. Compared to other registries, including a previous Korean registry, the cohort of patients with PAH in the HIRA database exhibited a similar distribution of age, sex, and survival rates. However, in terms of etiology, this cohort showed higher incidences of IPAH and CTD than other registries. Additionally, as class I is recommended in the PAH guidelines, the results of medication prescription distribution showed that most patients received monotherapy (CCB or bosentan).

## Supporting information

S1 Table Definitions and ICD-10 codes used for identifying comorbidities. (DOCX)

S2 Table Definitions and ICD-10 codes used for classifying the etiologies of acquired pulmonary arterial hypertension. (DOCX)

S3 Table Combined diagnoses in ICD codes I27.0 and I27.2. (DOCX)

S1 Fig Incidence trend of patients with pulmonary arterial hypertension. (TIF)
