# Supplementary material for: Demographics, treatment trends, and survival rate in incident pulmonary artery hypertension in Korea: A nationwide study based on the health insurance review and assessment service database
Source: PLoS One. 2018 Dec 19;13(12):e0209148. doi: 10.1371/journal.pone.0209148 (PMC6300275; doi:10.1371/journal.pone.0209148)
Supplement: S1 Table — (DOCX) [file pone.0209148.s001.docx]

**Supplementary Table 1.** Definitions and ICD-10 codes used for identifying comorbidities.

| **Comorbidities** | **ICD-10 codes** |
| --- | --- |
| Hypertension | I10, I11, I12, I13, I15 |
| Diabetes mellitus | E11, E12, E13, E14 |
| Ischemic heart failure | I20-I25 |
| Chronic kidney disease | N18, N19 |
| Liver cirrhosis | K74 |
| Arrhythmia | I48, I49 |
| Thyroid disease | E3, E5 |
| Previous TIA or stroke | ICD-10: G45.9 I60, I61, I62, I63, I64, I69 |
| History of malignancy | ICD-10: C00-C97 |

ICD-10, International Classification of Diseases-Tenth Revision; TIA, transient ischemic attack
