# Supplementary material for: Demographics, treatment trends, and survival rate in incident pulmonary artery hypertension in Korea: A nationwide study based on the health insurance review and assessment service database
Source: PLoS One. 2018 Dec 19;13(12):e0209148. doi: 10.1371/journal.pone.0209148 (PMC6300275; doi:10.1371/journal.pone.0209148)
Supplement: S2 Table — (DOCX) [file pone.0209148.s002.docx]

**Supplementary Table 2.** Definitions and ICD-10 codes used for classifying the etiologies of acquired pulmonary arterial hypertension

|  | **ICD-10 codes** |
| --- | --- |
| Connective tissue disease | M05, M06, M30, M31, M32, M33, M34, M35, M45, K76.5 |
| Congenital heart disease | I27.83  P29  Q20, Q21, Q22, Q23, Q24, Q25, Q26, Q27, Q28 |
| Others | B20, B21, B22, B23, B24  B65, K77  D55, D56, D57, D58, D59  K76.6 |

ICD-10, International Classification of Diseases-Tenth Revision
