# Supplementary material for: Demographics, treatment trends, and survival rate in incident pulmonary artery hypertension in Korea: A nationwide study based on the health insurance review and assessment service database
Source: PLoS One. 2018 Dec 19;13(12):e0209148. doi: 10.1371/journal.pone.0209148 (PMC6300275; doi:10.1371/journal.pone.0209148)
Supplement: S3 Table — (DOCX) [file pone.0209148.s003.docx]

**Supplementary Table 3.** Combined diagnoses in ICD codes I27.0 and I27.2

| ICD I27.0 |
| --- |
| Collagen vascular disease associated pulmonary arterial hypertension |
| Congenial systemic-to-pulmonary shunts associated pulmonary arterial hypertension |
| Drugs and toxins associated pulmonary arterial hypertension |
| Familial pulmonary arterial hypertension |
| HIV infection associated pulmonary arterial hypertension |
| Idiopathic pulmonary hypertension |
| Pulmonary arterial hypertension associated with collagen vascular disease |
| Pulmonary arterial hypertension associated with congenital systemic-to-pulmonary shunts |
| Pulmonary arterial hypertension associated with drugs and toxins |
| Pulmonary arterial hypertension |
| ICD I27.2 |
| Chronic thromboembolic pulmonary hypertension |
| Compression of pulmonary vessels (fibrosing mediastinitis, tumor, and adenopathy) |
| Exercise-induced pulmonary hypertension |
| Other secondary pulmonary hypertension |
| Other associated pulmonary arterial hypertension |
| Portal hypertension associated pulmonary arterial hypertension |
| Pulmonary arterial hypertension associated with others |
| Pulmonary capillary hemangiomatosis associated with significant venous or capillary involvement PAH |
| Pulmonary hypertension |
| Pulmonary hypertension associated with parenchymal lung disease and/or chronic hypoxemia |
| Pulmonary hypertension due to chronic thrombotic and/or embolic disease |
| Pulmonary hypertension due to congenital heart disease |
| Pulmonary hypertension due to miscellaneous conditions |
| Pulmonary veno-occlusive disease associated with significant venous or capillary involvement PAH |
| Pulmonary venous hypertension |
| Secondary pulmonary hypertension |

PAH, Pulmonary arterial hypertension; HIV, human immunodeficiency virus;
