# Supplementary figures and images for: Demographics, treatment trends, and survival rate in incident pulmonary artery hypertension in Korea: A nationwide study based on the health insurance review and assessment service database
Source: PLoS One. 2018 Dec 19;13(12):e0209148. doi: 10.1371/journal.pone.0209148 (PMC6300275; doi:10.1371/journal.pone.0209148)

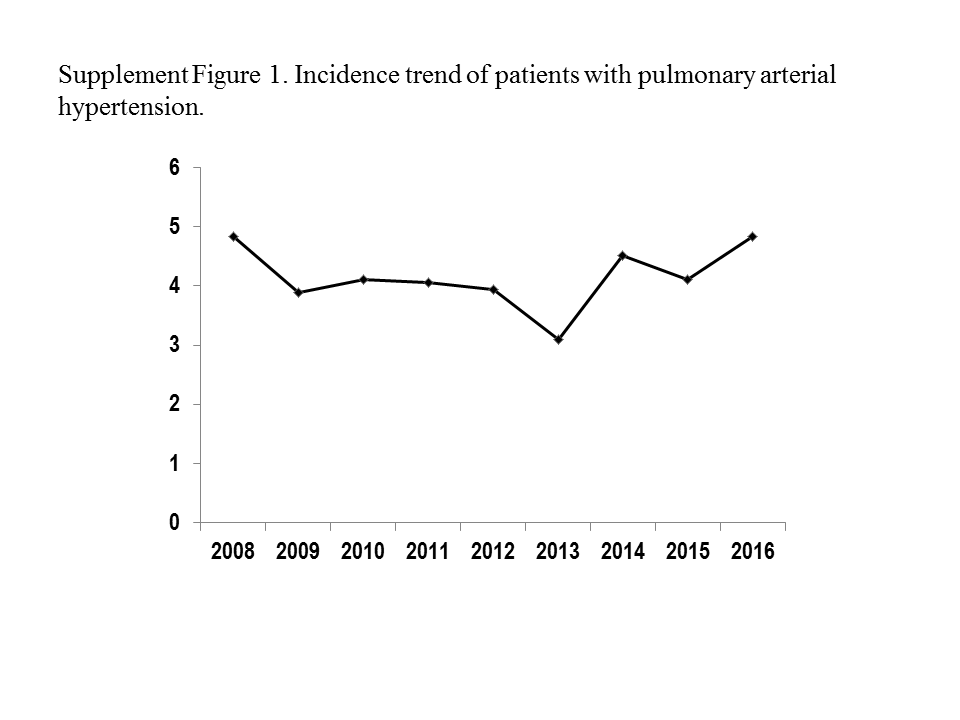

Supplement: S1 Fig — (TIF) [file pone.0209148.s004.tif]
